# Drug-related problems and medication reviews among old people with dementia

**DOI:** 10.1186/s40360-017-0157-2

**Published:** 2017-06-27

**Authors:** Bettina Pfister, Jeanette Jonsson, Maria Gustafsson

**Affiliations:** Department of Pharmacology and Clinical Neuroscience, Umeå University, SE-90187 Umeå, Sweden

**Keywords:** Clinical pharmacy, Medication reviews, Drug-related problems, Old people, Dementia

## Abstract

**Background:**

Drug-related problems, including medication errors and adverse drug events, are common among old people. Due to, for example, greater susceptibility to side effects, people with dementia are even more at risk of drug-related problems. The objectives of this study were to assess the occurrence and character of drug-related problems found among old people with dementia or cognitive impairment.

**Methods:**

Data from a randomized controlled clinical trial exploring the effects of a pharmacist intervention as part of a hospital ward team in patients 65 years and older with dementia or cognitive impairment were used. The study was conducted between 2012 and 2014 in the orthopedic and medicine wards in two hospitals located in Northern Sweden. Drug-related problems identified in this patient group were classified and described, and associations with different factors were investigated.

**Results:**

Clinical pharmacists identified at least one DRP in 66% (140/212) of participants in the intervention group, for a total of 310 DRPs. Ineffective drug/inappropriate drug and unnecessary drug therapy were the most common drug-related problems. Discontinuation of drug therapy was the most common action carried out. Drug-related problems were more common among people prescribed a larger number of drugs and among people with an earlier stroke.

**Conclusions:**

Drug-related problems are common among people with dementia and cognitive impairment. Comprehensive medication reviews conducted by clinical pharmacists as part of a health care team might be important to prevent, identify and solve these problems.

## Background

The use of drugs is a cornerstone of the care of older people. However, drug treatment in this group of people entails a significant risk of adverse drug events. Drug-related problems (DRPs) such as adverse drug reactions (ADRs), interactions, and potentially inappropriate drug use are common, and cause up to 30% of hospitalizations among old people [1]. Even more at risk are people with dementia or cognitive impairment, where 41% of hospital admissions have been judged as caused or partly caused by DRPs [2]. Age-related impairment of organic functions combined with other problems specific to this patient group, such as greater susceptibility to side effects and noncompliance, probably contributes to the increased rate of hospitalization [3–7]. Appropriate prescribing among old people is a considerable challenge, and optimizing drug therapy for old people with dementia is even more complex.

Clinical pharmacy can be defined as “a health specialty, which describes the activities and services of the clinical pharmacist to develop and promote the rational and appropriate use of medicinal products and devices” [8]. Clinical pharmacy can, for example, be an activity implemented in primary care or in a hospital setting, such as medication reconciliation or medication reviews [8]. Inadequate transfer of information at transition points between levels of care contributes to medication discrepancies, and medication reconciliation is performed in order to avoid this [9]. A medication review is a systematic evaluation of an individual patient’s medicine treatment. According to the Pharmaceutical Care Network Europe (PCNE), a medication review is defined as “an evaluation of a patient’s medicines with the aim of optimizing the outcomes of medicine therapy. This entails identifying the risks, detecting medication-related problems and suggesting solutions” [10].

Systematic reviews suggest that clinical pharmacist interventions can improve patient outcomes in both primary care or hospital settings across many countries; for example in the US, the clinical pharmacist’s role in the clinic has been established for several years [11, 12] In Sweden, studies concerning clinical pharmacy outcome have been few, and this has probably contributed to slower progress. However, in recent years, the number of clinical pharmacists working in hospital wards in Sweden has increased.

A recent study from Sweden showed that the addition of clinical pharmacists to the healthcare team did not reduce the risk of drug-related readmissions during a 180-days follow-up period, however, post-hoc and subgroup analyses indicated significant effects favouring the intervention [13]. In this study, the clinical pharmacists conducted medication reconciliation, medication reviews and participated in ward rounds, where clinically relevant DRPs were discussed with the health care team. The clinical pharmacists identified at least one DRP in 66% (140/212) of participants in the intervention group, for a total of 310 DRPs.

The objectives of the present study were to further assess the occurrence and character of DRPs found in the study above, as well as to describe the work with medication reviews. Another objective was to investigate associated factors to DRPs in old people with dementia or cognitive impairment.

## Methods

### Subjects and settings

Data for this study was gathered from a randomized controlled study (RCT) investigating the impact of adding the competence of a clinical pharmacist to a health care team [13]. Patients admitted to the acute internal medicine ward and to the orthopedic clinic at Umeå University Hospital, and patients from medicine wards at the County Hospital in Skellefteå were included. Both hospitals are located in Northern Sweden. Eligible patients were aged 65 years or older and had dementia or cognitive impairment. The patients were recruited between January 9, 2012 and December 02, 2014. In total, 460 patients, 65 years and older with dementia or cognitive impairment, were randomized to an intervention (230 persons) or control group (230 persons). In this study, only people in the intervention group were analyzed. The final sample was 212 persons (18 people died during index admission and were excluded).

### Intervention

Three clinical pharmacists, who were already part of the different ward teams at the time the study started, conducted the interventions. The additional service provided by the clinical pharmacists consisted of medication reconciliation, medication review, and participation in ward rounds.

A medication reconciliation was performed for all patients in order to find discrepancies between the medication chart at hospital admission, and what patients were actually taking (Table 1). However, uses of OTC drugs were not investigated in this study because the clinical pharmacists did not talk to the patients about their medications. A comprehensive medication review comprising aspects associated with the patient’s drug therapy was then performed by the clinical pharmacist (Table 1). The clinical pharmacists used all available data, including the medication list, list of laboratory results, medical record notes from primary care, as well as notes from earlier contacts with health care providers, to compile an extensive medication history. In addition, general data regarding age, gender, and patient history were collected. Clinical response to drug treatment was monitored throughout the hospital stay.

**Table 1:** Important aspects to consider when performing a medication review

*Impaired body function:* renal function, liver function, contraindications, allergies, swallowing problems *Certain drugs that need special attention:* toxic drugs, drugs prone to producing side effects, potentially inappropriate drugs *Interactions:* drug-drug, drug-food interactions, interactions between drugs and herbal medicinal products *The patient’s clinical symptoms in relation to drug treatment:* symptoms (adverse drug reactions) *Overall view of the patients’ medication:* proper drug selection, dosage, duration of treatment, polypharmacy, indication for therapy, untreated indication, compliance, OTC drugs, effectiveness, cost-effectiveness and general judgment of the patient’s drug use *Medication reconciliation:* by conducting medication reconciliation, the pharmacists ensure that the medication administration records used at the wards are updated, accurate, and complete. Various information sources should be used, including drug lists from primary care centers, the patients’ hospital medical records, and when possible, interviews with patients and/or relatives.	

*Abbreviations*: *OTC* over-the-counter

The clinical pharmacist participated in ward rounds, and clinically relevant DRPs were discussed with the health care team (physicians, nurses, enrolled nurses). Advice was given about drug selection, dosages, and possible monitoring needs. When a drug was considered inappropriate for some reason, alternative drugs were suggested. The attending physicians made the final decision concerning proposed changes to therapy. The acceptance or rejection of the pharmacist’s recommendation for changes in drug therapy was documented.

### Classifications of DRPs

DRPs were classified according to a modified version of Cipolle et al., [14] into seven subgroups: *ADR*, *dosage too high, dosage too low, ineffective drug*, *needs additional drug therapy, unnecessary drug therapy* and *noncompliance.* Inappropriate drugs were added to the category *ineffective drugs* and, additionally, three further categories were introduced: *interactions* (pharmacodynamic and pharmacokinetic), *transition errors* and *monitoring need*. These three extra categories were classified as ADRs in the main paper [13].

The DRPs were defined as follows:

*ADR:* Adequate doses resulting in adverse drug reactions were classified as ADR.

*Dosage too high:* If the prescribed dose was too high in relation to the patient’s renal function, liver function or age, this was classified as dosage too high.

*Dosage too low:* If the prescribed dose was less than recommended, this was classified as dosage too low.

*Ineffective/inappropriate drug*: Inappropriate drug use according to explicit Swedish criteria [15] and inappropriate drugs according to renal function or disease were classified as ineffective/inappropriate drug.

*Needs additional drug therapy:* If a patient was inadequately medicated, this was classified as needs additional drug therapy.

*Unnecessary drug therapy:* If a patient had an unnecessary drug therapy, this was classified as unnecessary drug therapy.

*Noncompliance:* A deviation from the prescribed medications because of a choice, non-comprehension or forgetfulness, was classified as noncompliance.

*Interactions:* A drug interaction was defined as the modification of one drug by concomitant administration of another drug, producing loss of therapeutic effect or too high therapeutic effect.

*Transition errors:* Discrepancies between the medication charts upon hospital admission as compared with what patients were actually taking

*Monitoring need:* Need for therapeutic drug monitoring, laboratory test.

### Data analysis

Simple logistic regression analyses were conducted to investigate the association between DRPs and different factors extracted from the medical record. These factors were gender, age, number of medications, type of ward, type of living, Mini Mental State Examination (MMSE), creatinine clearance and the patients’ medical histories. A multiple logistic regression analysis was conducted including significant variables from the simple models.

Results are presented as odds ratios (ORs) with 95% confidence intervals (CIs). *P*-values < 0.05 were considered statistically significant. All analyses were conducted using the Statistical Package for the Social Sciences (SPSS) for Windows, Version 23.0.

## Results

Of the 212 people included in the study, 133 (62.7%) were women and the mean age was 83.1 ± 6.6 years. Alzheimer’s disease [64/212 (30.2%)] and vascular dementia [42/212 (19.8%)] were the most common dementia diagnoses. The remaining 106 people suffered from Lewy body dementia [5/212 (2.4%)], unspecified dementia, or cognitive impairment [101/212 (47.6%)], according to the medical record. Most patients [146/212 (68.9%)] lived at home.

Among the 310 DRPs identified by the clinical pharmacists in 66% (140/212) of the participants, ineffective drug/inappropriate drug (*n* = 54) and unnecessary drug therapy (*n* = 54) were the most common, followed by dosage too high (*n* = 44). Further DRPs were classified as ADR (*n* = 41), needs additional drug therapy (*n* = 37), transition error, (*n* = 26), interactions (*n* = 23), dosage too low (*n* = 14), monitoring need (*n* = 13) and noncompliance (*n* = 4) (Table 2).

**Table 2:** Clinically relevant DRPs identified by clinical pharmacists and discussed with the health care team

Type of DRPs	Identified problem/potential problem	Identified no. of DRPs (no. acted upon)	Drugs involved (frequency)
Suspected adverse drug reaction (*n* = 41)	Acute renal failure	1 (1)	Irbesartan
	Anemia	1 (0)	Acetylsalicylic acid
	Confusion	3 (3)	Metoprolol, morphine, solifenacin
	Fall	7 (5)	Alfuzosin, candesartan, citalopram, mirtazapine, oxazepam, zopiclone (2)
	Fatigue	5 (4)	Mianserin, morphine (2), olanzapine, propiomazine
	Hallucinations	1 (1)	Citalopram
	Hypercalcemia	1 (1)	Bendroflumethiazide
	Hyperkalemia	1 (1)	Spironolactone
	Hypochloremia, high CO_2_	1 (1)	Furosemide
	Hypokalemia	1 (1)	Furosemide
	Hyponatremia	2 (1)	Buspirone, losartan
	Hypotension	2 (2)	Alfuzosin (2)
	Increased INR	1 (0)	Avlosulfon
	Liver disorder	2 (2)	Simvastatin (2)
	Nausea	4 (3)	Codeine/acetaminophen, galantamine, levetiracetam, metformin
	Edema	1 (1)	Amlodipine
	Orthostatic hypotension	2 (1)	Bendroflumethiazide, isosorbide mononitrate
	Seizure	1 (0)	Donepezil
	Sleeping problems	1 (1)	Bisoprolol
	Thrombocytopenia	1 (1)	Valproic acid
	Urinary retention	2 (2)	Amitriptyline, citalopram
Dosage too high (44)	Dosage too high according to indication/guidelines	18 (15)	Acetylsalicylic acid (5), allopurinol, citalopram (2), folic acid (4), metoclopramide, omeprazole, risperidone, trihexyphenidyl^a^ (2), zuclopenthixol
	Dosage too high according to patient response	10 (8)	Bisoprolol, clomethiazole, furosemide (2), isosorbide mononitrate, levothyroxine (2), mirtazapine, potassium, spironolactone
	Dosage too high according to liver function	1 (1)	Acetaminophen
	Dosage too high according to renal function	12 (12)	Allopurinol, digoxin (3), enalapril, fondaparinux, glipizide, memantine (3), metformin, sucralfate
	Dosage too high according to maximum dose per day	2 (2)	Acetaminophen (2)
	Lack of gradual dose increase	1 (1)	Rivastigmine
Dosage too low (14)	Dosage too low according to indication/guidelines	14 (14)	Acetylsalicylic acid^b^, amoxicillin (2), calcium (2), dalteparin, ferrous succinate, flucloxacillin, ipratropium, losartan, omeprazole (2), pivecillinam, sodium picosulfate^c^
Ineffective/Inappropriate drug (54)	Inappropriate drug according to cost	4 (4)	Escitalopram, oxycodone (2), pregabalin
	Inappropriate drug according to renal function	12 (12)	Glibenclamide (2), hydrochlorothiazide, ibumetin, ketoprofen, metformin (2), morphine (2), nitrofurantoin, tramadol (2)
	Inappropriate drug according to liver function	1 (1)	Clomethiazole
	Inappropriate drug according to guidelines	4 (3)	Acetylsalicylic acid/dipyramidole, lactitol, methenamine hippurate, oxycodone + buprenorphine
	Drugs that should be avoided in the elderly	17 (10)	Amitriptyline, diazepam, fesoterodine, flunitrazepam, haloperidol, hydroxyzine, propiomazine (3), solifenacin, tolterodine (2), tramadol, triazolam, zolpidem (3)
	Heart failure	6 (6)	Acetaminophen soluble tablet qd, acetylcysteine soluble tablet qd, diclofenac, ibuprofen, naproxen, potassium^d^
	Atrial fibrillation	1 (0)	Propranolol
	Hyperkalemia	2 (2)	Potassium IV (2)
	Hypertension	4 (3)	Acetaminophen soluble tablet qd (2), atenolol, pindolol
	Myocardial infarction, past	1 (1)	Medroxyprogesterone acetate
	Palpitations	1 (1)	Metoprolol sustained-release tablet
	Risk in this specific patient group	1 (0)	Codeine/acetaminophen + acetaminophen PRN
Interaction (23)	Interactions	23 (16)	Alendronat + calcium
			Calcium + ciprofloxacin
			Carbamazepine + citalopram
			Carbamazepine + doxycycline
			cholestyramine (PRN) + Warfarin + levothyroxine + furosemide
			Doxycycline + calcium
			Ferrous glycine sulfate + levothyroxine
			Ferrous glycine sulfate + levodopa/benserazide
			Ferrous succinate + calcium + levothyroxine
			Ferrous succinate + calcium (4)
			Ferrous succinate + ciprofloxacin
			Ferrous succinate + levothyroxine (2)
			Levothyroxine + magnesium hydroxide
			Levothyroxine + magnesiumhydroxide + calcium
			Warfarin + diclofenac (2)
			Warfarin + ginkgo biloba
			Warfarin + prednisolone
			Warfarin + citalopram
Monitoring need (13)	Lack of liver function tests^e^	1 (1)	Acetaminophen
	Lack of serum digoxin test	1 (1)	Digoxin
	Lack of serum hemoglobin A_1c_ test	1 (1)	
	Lack of serum homocysteine test	1 (1)	Vitamin B combination
	Lack of serum potassium test	2 (2)	Spironolactone (2)
	Lack of serum potassium and serum creatinine tests	1 (1)	Enalapril and spironolactone
	Lack of serum uric acid test	1 (1)	Diazoxide
	Lack of thyroid function tests	4 (4)	Levothyroxine (4)
	Lack of thyroid function tests^f^	1 (1)	
Needs additional drug therapy/untreated/undertreated indication (37)	Asthma	1 (1)	Short-acting inhaled beta-2-agonist
	Heart failure	8 (6)	ACE-inhibitor (4), beta-blocker (2), spironolactone (2)
	Hypertension	1 (1)	ACE-inhibitor
	Hypokalemia	1 (1)	Potassium
	Increased risk of obstipation	7 (5)	Opioids without laxantia (7)
	Increased risk of ulcus	5 (4)	Clopidogrel, acetylsalicylic acid + galantamine without PPI
			Prednisolone + acetylsalicylic acid + donepezil without PPI
			Prednisolone + acetylsalicylic acid without PPI
			Warfarin + prednisolone without PPI
			Previous ulcus, PPI discontinued by mistake
	Myocardial infarction, past	2 (0)	Beta-blocker, acetylsalicylic acid
	Osteoporosis/vertebral compression fracture	3 (2) 1 (1)	Calcium/vitamin D3 (3) Bisphosphonate
	Pain	1 (1)	Acetaminophen
	Seizure	1 (1)	Gabapentin^g^
	Stroke, past	3 (2)	Anticoagulant (3)
	Stroke, past	1 (0)	Statin
	TIA, past	1 (1)	Anticoagulant
	Wernicke-Korsakoff syndrome	1 (1)	Vitamin B combination
Noncompliance (4)	Handling problems – crushing	1 (0)	Doxazosin sustained-release tablet, hydroxycarbamide, metoprolol sustained-release tablet, morphine sustained-release tablet, omeprazole^h^
	Handling problems – inhalation technique	2 (2)	Budesonide, terbutaline^h^ Budesonide, indacaterol, terbutaline, tiotropium^h^
	Overuse	1 (0)	Hydroxyzine
Transition error (26)	Wrong dose or time of dose in the medical record	11 (9)	Citalopram, digoxin, hydralazine, mianserin(2), mirtazapine, pramipexole, risperidone, vitamin B, zolpidem, zopiclone
	Drug incorrectly registered in the medical record	6 (6)	Ciprofloxacin, fluconazol^i^, metformin, metoprolol, mianserin, simvastatin
	Drug is missing in the medical record	8 (7)	Acetaminophen (2), acetylsalicylic acid, bimatoprost/timolol, citalopram, ibuprofen, levothyroxine, memantine,
	Wrong information about the drug in the medical record	1 (0)	Ketobemidone
Unnecessary drug therapy (54)	No indication for drug use	48 (41)	Alendronat, allopurinol, amlodipine (2), bendroflumethiazide, carbamazepine, citalopram, clemastine, codeine/acetaminophen, cyanocobalamin/folic acid/pyridoxine hydrochloride, enalapril, ferrous succinate (2), folic acid, folic acid/cyanocobalamin (2), furosemide (5), gabapentin, haloperidol (2), ibumetin, loperamide, losartan, magnesium hydroxide (3), metformin (4), metoprolol, acetaminophen, potassium(4), prednisolone, probenecide, ranitidine, simvastatin, sodium hydrogen carbonate (2), zopiclone (2)
	Inappropriate duplication	6 (6)	Estradiol vaginal ring + estradiol vaginal tablet
			lactulose + macrogol/electrolytes (3)
			warfarin + acetylsalicylic acid^j^
			warfarin + clopidogrel^k^

*Abbreviations*: *DRP* drug-related problem, *LMWH* low molecular weight heparin, *PPI* proton pump inhibitors, *PRN* Pro Re Nata, *TIA* transient ischemic attack

^a^Dose of antipsychotic lowered, but not trihexyphenidyl (Table 3)

^b^The patient had atrial fibrillation

^c^Prescribed PRN

^d^Spironolactone suggested

^e^The patient was overusing acetaminophen

^f^The patient had atrial fibrillation

^g^Gabapentine discontinued as doctors thought the indication was pain; really, it was epilepsy

^h^Classified as one DRP

^i^Fluconazol in the category interaction (with citalopram) in Table 3

^j^Warfarin prescribed instead of acetylsalicylic acid, both treatments continued by mistake

^k^Warfarin prescribed instead of clopidogrel, both treatments continued by mistake

More detailed examples of DRPs identified among these patients are listed in Table 3.

**Table 3:** Examples of DRP

Type of DRP	Comment
Adverse drug reaction	A 78-year-old man with Alzheimer’s disease, hypertension and hypokalemia was admitted to the hospital because of hypertension (205/115 mmHg). The doctor initially suspected that the patient’s symptom was an ADR related to galantamine, and so discontinued the treatment. However, hypertension secondary to primary aldosteronism was then diagnosed, and ten days later galantamine was restarted at the same dosage as at admission (i.e., 24 mg daily). The patient was ready to be discharged, but got nauseous and vomited and had to stay on the ward. The clinical pharmacist suspected an ADR and suggested to decrease the dose of galantamine, which was done. The symptoms resolved and the patient could be discharged.
Dosage too high	A 71-year-old man was admitted to the hospital because of a history of falls. His chronic medical problems included schizophrenia, diabetes mellitus type II, mental retardation and a suspected dementia. He had a catheter because of urinary retention. A UTI was diagnosed. His schizophrenia was treated with zuclopenthixol decanoate intramuscular injections every fourth week, and for side effects with trihexyphenidyl 20 mg daily. The dosage of zuclopenthixole had been lowered by more than 75% over recent years whilst the dosage of trihexyphenidyl was unchanged. The clinical pharmacist questioned the dose of the anticholinergic drug that might have been a contributory factor to suspected dementia, history of falls and urinary retention. The dose of trihexyphenidyl was gradually lowered and finally discontinued, and the injection switched to risperidone tablets.
Dosage too low	An 89-year-old man with cognitive impairment was admitted to the hospital because of urosepsis. His medical history included stroke and abdominal pain, which was treated with sustained-release morphine 30 mg twice daily, and sodium picosulfate PRN for prevention of opioid-associated constipation. Examination on the ward revealed severe constipation, which was treated with methylnaltrexone. The patient’s MMSE score several weeks before hospital admission was 13/30. Because of his low MMSE score and the fact that he was living at home on his own, it was unclear whether the patient understood the importance/need of taking the laxative in time. The clinical pharmacist suggested regular dosing of sodium picosulfate, a recommendation that was followed by the physician. Osmotic laxatives were also prescribed.
Ineffective/Inappropriate drug	A 90-year-old woman with cognitive impairment was admitted to the hospital because of excessive daytime sleepiness. A medication review performed by the clinical pharmacist revealed that medication with propiomazine 25 mg at bedtime was started by primary care 8 days prior to admission to the hospital. Propiomazine can cause daytime sleepiness and is classified as an inappropriate drug by the quality indicator developed by the Swedish National Board of Health and Welfare. Propiomazine was discontinued.
Interaction	An 86-year-old man with Alzheimer’s disease was admitted to the hospital because of bursitis. Two months before admission, he was prescribed fluconazole 50 mg daily as a seven-day treatment, but due to a transcription error, it was added to the medication list as an ongoing prescription. The patient also had an ongoing treatment with citalopram. On the ward, he got more and more agitated, and hallucinated. Haloperidol was prescribed. The patient’s symptoms might have been a result of increased concentrations of citalopram due to an interaction between citalopram and fluconazole. The clinical pharmacist recommended discontinuation of fluconazole and haloperidol. Fluconazole was discontinued and haloperidol was prescribed PRN, and since the hallucinations disappeared, haloperidol was no longer needed.
Needs additional drug therapy	An 87-year-old woman was admitted to the hospital because of deteriorating heart failure. She had a medical history of atrial fibrillation, angina pectoris, heart failure, stroke and vascular dementia, with an MMSE score of 14/30. She was agitated and aggressive to the staff and it was assumed that she suffered from pain, which was treated with oxycodone PRN. A medication review performed by the clinical pharmacist revealed that gabapentin was discontinued for unclear reasons just a week prior to admission to the hospital. The indication for gabapentin use was not only neuropathic pain but also post-stroke epilepsy, of which the physician was unaware. Gabapentin was the only antiepileptic drug treatment the patient had been prescribed. Gabapentin was reinitiated.
Noncompliance	One patient admitted to the ward for dyspnea had been prescribed a multidrug treatment for COPD (stage III) with dry powder inhalers. According to the medical record, the patient required full support to cope with activities of daily living and could not follow instructions. It is therefore possible that the patient was unable to use the inhaler devices properly prior to readmission, leading to ineffective drug treatment. The pharmacist recommended the use of a pressurized metered-dose inhaler together with a spacer instead.
Unnecessary drug therapy	An 89-year-old woman with vascular dementia, diabetes mellitus, previous stroke and angina pectoris was admitted to the hospital because of headache and abnormal motor function; meningitis was diagnosed. The patient was also nauseous and had been so for a long time. In 2005, she had been prescribed haloperidol for the treatment of her nausea, and she was still treated with this at the time of admission (2012). Her diabetes was treated with metformin, which could be the cause of her nausea. Because of decreased renal function and an HbA1c fluctuating between 46–58 mmol/mol during the last two years, the clinical pharmacist suggested that both haloperidol and metformin should be discontinued (with monitoring of HbA1c later on), which was done.

*Abbreviations*: *ADR* adverse drug reaction, *COPD* chronic obstructive pulmonary disease, *DRP* drug-related problem, *MMSE* Mini Mental State Examination, *PRN* Pro Re Nata, *UTI* urinary tract infection

Suggested actions were carried out for 82%, where discontinuation of drug therapy was the most common (*n* = 78), followed by the category “other” which includes, for example, monitoring of laboratory values or correction of transition errors (*n* = 55). Other actions were reduction in dosage (*n* = 45), initiation of drug therapy (*n* = 21), change of drug (*n* = 19), increase in dosage (*n* = 8) and change of drug formulation (*n* = 4). Further, 24 suggestions were written in discharge notes, and 56 of the suggestions were rejected.

DRPs were more common among people taking a higher number of drugs (OR, 1.255 [95% CI, 1.137-1.385]) and among people with an earlier stroke (OR, 5.042 [95% CI, 2.032-12.509]). There were no significant differences between patients with and without DRPs regarding gender, living arrangement, or ward type (Table 4). In a multivariate model with DRP as the dependent variable and significant variables from the simple model as independent variables (number of drugs at admission, age [borderline significant], and stroke), the number of drugs (OR, 1.241 [95% CI, 1.122-1.374]) and stroke (OR, 4.306 [95% CI, 1.685-11.005]) remained significant.

**Table 4:** Characteristics of study population with and without DRP

	People with DRPs *N =* 140	People without DRP *n* = 72	Simple OR (95% CI)	Multiple OR (95% CI)
Cases *n* (%)	140 (66.0)	72 (33.9)		
Women, *n* (%)	88 (62.9)	45 (62.5)	1.015 (0.564-1.827)	
Age mean ± SD	83.7 ± 6.6	82.0 ± 6.3	1.042 (0.997-1.088)	1.041 (0.994-1.090)
Number of drugs at randomization ± SD	9.3 ± 3.4	6.8 ± 3.4	1.255 (1.137-1.385)	1.241 (1.122-1.374)
Type of ward				
Orthopedic ward *n* (%)	20 (14.3)	9 (12.5)	ref	
Medical ward *n* (%)	120 (85.7)	63 (87.5)	0.857 (0.369-1.993)	
Type of living				
Living at home *n* (%)	91 (65.0)	55 (76.4)	ref	
Nursing home *n* (%)	49 (35.0)	17 (23.6)	1.742 (0.914-3.321)	
MMSE (0–30) ± SD	18.9 ± 4.8	20.7 ± 4.5	0.923 (0.842-1.013)	
Creatinine clearance (mL/min)	52.8 ± 22.3	55.0 ± 21.1	0.995 (0.983-1.008)	
Medical history				
Heart failure *n* (%)	50 (35.7)	22 (30.6)	1.263 (0.687-2.322)	
Cardiac arrhythmia *n* (%)	40 (28.6)	22 (30.6)	0.909 (0.488-1.692)	
Diabetes mellitus *n* (%)	43 (30.7)	18 (25.0)	1.330 (0.699-2.530)	
Chronic obstructive pulmonary disease *n* (%)	10 (7.1)	6 (8.3)	0.846 (0.295-2.429)	
Stroke, past *n* (%)	44 (31.4)	6 (12.0)	5.042 (2.032-12.509)	4.306 (1.685-11.005)

*Abbreviations*: *CI* confidence interval, *DRP* drug-related problem, *MMSE* Mini Mental State Examination (*n* = 157), *OR* odds ratio, *SD* standard deviation. Creatinine clearance was based on P-creatinine applying the Cockcroft-Gault equation. The multivariate model includes significant variables as independent variables; number of medications at randomization, stroke and age (borderline significant)

## Discussion

The frequency of potential DRPs in old people with dementia or cognitive impairment was high in this study, which is in line with or slightly lower than in previous research performed among old people, though not specifically among people with dementia [16, 17]. Ineffective drug/inappropriate drug and unnecessary drug therapy were the most frequent DRPs. Discontinuation of drug therapy was the most common action carried out. DRPs were more common among people prescribed a larger number of drugs and among people with an earlier stroke.

To be able to conduct comprehensive medication reviews with good quality, the clinical pharmacists need full access to medical and laboratory records. In contrast to many other health care systems, in the county of Västerbotten, primary and hospital care use the same electronic medical record system making it possible to see data from medical record notes from different care settings which is needed to compile an extensive medication history. This is important, for example, to understand why a drug is prescribed from the beginning or to find out any earlier adverse effects. It also makes it possible to see patients’ drug lists from both primary care and hospital care which facilitates the potential of obtaining an accurate medication list, which is essential for assessing the patient’s medication treatment. However, discrepancies were still found in the present study, some of which could have resulted in patient harm; for example, there was an incorrect digoxin dosage prescribed at admission to the ward. Discrepancies in admission and discharge medications have also been found in previous research [18].

In a medication review, there are many aspects regarding drug therapy that need to be considered. Age-related changes in pharmacokinetics and pharmacodynamics in older people such as renal and liver impairment may increase the risk of side effects [19]. Inappropriate drugs due to impaired renal function are prevalent among old people according to previous research, [20] as well as among old people with dementia or cognitive impairment [21]. Examples of drugs prescribed inappropriately in patients with impaired renal function in the present study were oral antidiabetics such as metformin and glibenclamide. Among dosage too high, gabapentin and allopurinol were examples of drugs not dose-adjusted to the patients’ renal function. Clomethiazole was inappropriately prescribed for a patient with liver impairment, which could increase the bioavailability of clomethiazole significantly, leading to hypotension and falls [22]. Contraindications, allergies, and swallowing problems are other potential drug-related problems that need to be identified in order to prevent inappropriate prescriptions.

It is important to pay attention to drugs known to cause problems for patients. Belonging to this group are, for example, drugs with a narrow therapeutic index, such as digoxin and lithium, or drugs that might give serious adverse reactions, such as warfarin or methotrexate. Potentially inappropriate drugs according to guidelines [15] should also be identified in a medication review, since these drugs have been associated with hospitalizations and higher mortality [2, 23]. Still, inappropriate drugs are prevalent among old people with dementia, and are probably prescribed in many cases to treat behavioral and psychological symptoms despite known side effects and limited effect [24]. According to the Swedish National Board of Health and Welfare, [15] potentially inappropriate drugs found in the present study were, for example, long-acting benzodiazepines, antipsychotics and anticholinergic drugs. Additionally, another drug class belonging to potentially inappropriate drugs, non-steroidal anti-inflammatory drugs (NSAIDs), was prevalent among some of the patients in spite of concomitant heart failure and renal failure, which is surprising. It is well known that old people are at high risk of developing side effects from NSAIDs, such as gastrointestinal bleeding and renal toxicity, and that NSAIDs also increase the risk of hypertension and heart failure [25, 26].

Further, drug-drug interactions and interactions between drugs and herbal medicinal products are important to consider, since these can lead to adverse drug events and can be the reason for hospital admissions [2, 27]. In primary care, as well as today in the hospital care setting in Västerbotten County, prescribers have access to an interaction module in the electronic medical record system that is intended to identify and prevent important interactions. Nevertheless, important interactions were found that had not been acted upon such as, for example, interactions caused by drugs containing di- and trivalent cations such as calcium, magnesium and iron. Antibiotics like ciprofloxacin and doxycycline, as well as levothyroxine and bisphosphonates, are drugs whose absorption is reduced by these cations if administered simultaneously per os [28–30].

Many DRPs were classified as “needs additional drug therapy.” One of the most common DRPs in this group was people with opioids prescribed without concomitant laxatives and under-prescription of drugs for heart failure. Untreated conditions and under-prescriptions of beneficial medicines in older people are important to identify, probably even more important among people with dementia, since executive dysfunction may lead to difficulties in identifying, recognizing, and reporting adverse drug events. A side effect that may be relatively easy to handle normally, such as constipation, may become a major problem in patients with dementia, possibly leading to hospitalization if recognition is delayed.

Whether the indication for a therapy still exists is also an important factor to investigate when performing a medication review in order to avoid unnecessary drug events. There might be several reasons, but mechanistic renewal of prescriptions is probably one reason for why people have the same medications for prolonged times. Antihypertensive medications, oral iron formulations and antihistamines without current indication are examples from the present study.

Noncompliance accounted for only 4/310 (1.3%) of the DRPs found in this study, a number that is probably greatly underestimated. Because the patients suffered from dementia or cognitive impairment, the clinical pharmacists did not talk to them and, therefore, could not assess compliance. Even a small degree of cognitive impairment may have major negative impact on compliance with drug therapy among the healthy elderly, [7] and this can, for example, mean that the patients might be unable to use inhaler devices properly, leading to ineffective drug treatment. In addition, uses of OTC drugs were not investigated in this study. Many existing DRPs were probably not discovered in the present study.

DRPs were more common among people prescribed a larger number of drugs in this study. A larger number of drugs have also been associated with an increased risk of hospitalization among people with dementia and cognitive impairment [2]. However, it is also important to evaluate drug lists with only a few drugs. In one study, pharmacist intervention appeared to be more effective in preventing visits to the ED in patients taking <5 drugs on admission than in those taking ≥5 drugs [31].

DRPs were also more common among people with an earlier stroke. The reason for this could possibly be due to the use of secondary preventive drugs among this group of people such as antiplatelet agents, anticoagulant and hypertensive drugs. In previous studies, cardiovascular drugs have been associated with adverse drug events such as hypotension and electrolyte disturbances, and antiplatelet and anticoagulant drugs with haemorrhage, [32] which likewise was the case in the present study. However, a lack of secondary preventive drugs among people with a previous stroke also gave rise to potential DRPs discussed with the ward team in the present study.

The most common action taken for the clinical pharmacists’ suggestions was discontinuation of drug therapy. Another action was reduction in dosage due to, for example, impaired renal function. Suggestions were only rejected in 18% of the cases; hence, the acceptance rate was high. This is probably due to the fact that clinical pharmacists were already part of and accepted by the ward team at the start of the study. In addition, adequate training in clinical pharmacy and experience are important factors for being prepared to meet the specific challenges of clinical pharmacy work in primary care and in hospital care.

Some limitations of this study have to be taken into account. Since the clinical pharmacists did not talk to the patients, it could not be assessed whether the medications were actually taken by the patient. In addition, uses of OTC drugs were not investigated in this study. Further, whether the DRPs identified by the clinical pharmacists were clinically relevant and significant was not evaluated. However, based on the high acceptance rate (82%), it is reasonable to assume that most of the DRPs were judged to be clinically relevant by the physician in charge.

## Conclusion

Drug-related problems are common among people with dementia and cognitive impairment. Comprehensive medication reviews conducted by clinical pharmacists as part of a health care team might be important for preventing, identifying and solving these problems.

## Abbreviations

ADR: Adverse drug reaction
DRP: Drug related problem
MMSE: Mini Mental State Examination
NSAID: Non-steroidal anti-inflammatory drugs
OTC: Over-the-counter
PCNE: Pharmaceutical Care Network Europe
RCT: Randomized controlled study
SPSS: Statistical package for the social science

## References

[1] Chan M, Nicklason F, Vial JH (2001). Adverse drug events as a cause of hospital admission in the elderly. Intern Med J.

[2] Gustafsson M, Sjölander M, Pfister B, Jonsson J, Schneede J, Lövheim H (2016). Drug-related hospital admissions among old people with dementia. Eur J Clin Pharmacol.

[3] Turnheim K (2004). Drug therapy in the elderly. Exp Gerontol.

[4] Moore A, O’Keeffe S (1999). Drug- Induced Cognitive Impairment in the Elderly. Drugs Aging.

[5] Krista LL, Nathan H, Goran E, Van Robert R, Kenton R, Claudio AN (2002). Central Serotonergic Activity is Related to the Aggressive Behaviors of Alzheimer's Disease. Neuropsychopharmacology.

[6] Mehta D, Short J, Hilmer S, Nicolazzo J (2015). Drug Access to the Central Nervous System in Alzheimer’s Disease: Preclinical and Clinical Insights. Pharm Res.

[7] Hayes TL, Larimer N, Adami A, Kaye JA (2009). Medication Adherence in Healthy Elders. J Aging Health.

[8] ESCP. European Society of Clinical Pharmacy. http://www.escpweb.org. Accessed 14 Jan 2016.

[9] Scullin C, Scott MG, Hogg A, McElnay JC (2007). An innovative approach to integrated medicines management. J Eval Clin Pract.

[10] PCNE. Pharmaceutical Care Network Europe. Statement on medication review 2011–2012. http://www.pcne.org. Accessed 23 Nov 2015.

[11] Graabaek T, Kjeldsen LJ (2013). Medication reviews by clinical pharmacists at hospitals lead to improved patient outcomes: a systematic review. Basic Clin Pharmacol Toxicol.

[12] Chisholm-Burns MA, Kim Lee J, Spivey CA (2010). US pharmacists' effect as team members on patient care: systematic review and meta-analyses. Med Care.

[13] Gustafsson M, Sjölander M, Pfister B, Jonsson J, Schneede J, Lövheim H. Pharmacist participation in hospital ward teams and hospital readmission rates among people with dementia: a randomized controlled trial. Eur J Clin Pharmacol. 2017;73:827–35. 10.1007/s00228-017-2249-8 PMC5486919 28391409

[14] Cipolle R, Strand L, Morely P (1998). Pharmaceutical Care Practice.

[15] The National Board of Health and Welfare (2010). Indikatorer för god läkemedelsterapi hos äldre. Eng. Indicators for evaluating the quality of older people's drug therapy.

[16] Blix H, Viktil K, Moger T, Reikvam Å (2006). Characteristics of drug- related problems discussed by hospital pharmacists in multidisciplinary teams. Pharm World Sci.

[17] Gillespie U, Alassaad A, Henrohn D (2009). A comprehensive pharmacist intervention to reduce morbidity in patients 80 years or older: a randomized controlled trial. Arch Intern Med.

[18] Bishop MA, Cohen BA, Billings LK, Thomas EV (2015). Reducing errors through discharge medication reconciliation by pharmacy services. Am J Health Syst Pharm.

[19] Mangoni AA, Jackson SH (2004). Age-related changes in pharmacokinetics and pharmacodynamics: basic principles and practical applications. Br J Clin Pharmacol.

[20] Khanal A, Peterson GM, Castelino RL, Jose MD (2015). Potentially inappropriate prescribing of renally cleared drugs in elderly patients in community and aged care settings. Drugs Aging.

[21] Sönnerstam E, Sjölander M, Gustafsson M. Inappropriate Prescription and Renal Function Among Older Patients with Cognitive Impairment. Drugs & Aging. 2016;33:889–99. 10.1007/s40266-016-0408-8 PMC5122609 27734278

[22] Semla TP, Beizer JL, Higbee MD (2011). Geriatric Dosage Handbook.

[23] Gosch M, Wortz M, Nicholas JA, Doshi HK, Kammerlander C, Lechleitner M (2014). Inappropriate prescribing as a predictor for long-term mortality after hip fracture. Gerontology.

[24] Gustafsson M, Sandman PO, Karlsson S, Gustafson Y, Lovheim H (2013). Association between behavioral and psychological symptoms and psychotropic drug use among old people with cognitive impairment living in geriatric care settings. Int Psychogeriatr.

[25] Langman MJ, Weil J, Wainwright P (1994). Risks of bleeding peptic ulcer associated with individual non-steroidal anti-inflammatory drugs. Lancet.

[26] Bleumink GS, Feenstra J, Sturkenboom MC, Stricker BH (2003). Nonsteroidal anti-inflammatory drugs and heart failure. Drugs.

[27] Juurlink DN, Mamdani M, Kopp A, Laupacis A, Redelmeier DA (2003). Drug-drug interactions among elderly patients hospitalized for drug toxicity. JAMA.

[28] Kara M, Hasinoff B, McKay D, Campbell N (1991). Clinical and chemical interactions between iron preparations and ciprofloxacin. Br J Clin Pharmacol.

[29] Ogura Y, Gonsho A, Cyong J-C, Orimo H (2004). Clinical trial of risedronate in Japanese volunteers: a study on the effects of timing of dosing on absorption. J Bone Miner Metab.

[30] Campbell NR, Hasinoff BB, Stalts H, Rao B, Wong NC (1992). Ferrous sulfate reduces thyroxine efficacy in patients with hypothyroidism. Ann Intern Med.

[31] Alassaad A, Bertilsson M, Gillespie U, Sundstrom J, Hammarlund-Udenaes M, Melhus H (2014). The effects of pharmacist intervention on emergency department visits in patients 80 years and older: subgroup analyses by number of prescribed drugs and appropriate prescribing. PLoS One.

[32] Salvi F, Marchetti A, D'Angelo F, Boemi M, Lattanzio F, Cherubini A (2012). Adverse drug events as a cause of hospitalization in older adults. Drug Saf.

